# Sorafenib Restores Pentose Phosphate Pathway‐Related Redox Homeostasis via the c‐Raf/HSP90/G6PD Axis in Hepatic Ischemia‐Reperfusion Injury

**DOI:** 10.1002/mco2.70819

**Published:** 2026-06-21

**Authors:** Fengqiang Gao, Libin Dong, Yawen Tan, Zhen Zhang, Shengjun Xu, Zijian Lou, Yichao Wu, Siyu Chen, Li Zhuang, Zhengxing Lian, Shusen Zheng, Nasha Qiu, Kai Wang, Xiao Xu

**Affiliations:** ^1^ Hepatobiliary Center the First Aﬃliated Hospital with Nanjing Medical University Nanjing China; ^2^ Institute of Translational Medicine Zhejiang University School of Medicine Hangzhou China; ^3^ Department of Orthopedics The First Affiliated Hospital of Zhejiang University School of Medicine Hangzhou China; ^4^ Department of Hepatobiliary & Pancreatic Surgery Xihu University School of Medicine Affiliated Hangzhou First People's Hospital Hangzhou China; ^5^ General Surgery, Cancer Center, Department of Hepatobiliary & Pancreatic Surgery and Minimally Invasive Surgery Zhejiang Provincial People's Hospital (Affiliated People's Hospital) Hangzhou Medical College Hangzhou China; ^6^ Department of Hepatobiliary and Pancreatic Surgery Shulan (Hangzhou) Hospital Hangzhou China

**Keywords:** liver ischemia‐reperfusion injury, pentose phosphate pathway, redox homeostasis, sorafenib

## Abstract

Hepatic ischemia reperfusion injury (IRI) is a frequent complication of liver surgery and is strongly associated with poorer recipient survival. Sorafenib, a multi‐kinase inhibitor, has been implicated in hepatic metabolic and redox regulation, yet its role in hepatic IRI remains unclear. Our study finds that middle‐dose sorafenib protects the liver from IRI by reducing hepatic necrosis, inflammation, and apoptosis. Mechanistically, transcriptomic and metabolomics analyses confirm that middle‐dose sorafenib enhances pentose phosphate pathway‐mediated antioxidant activity through the c‐rapidly accelerated fibrosarcoma (c‐Raf)/heat shock protein 90 (HSP90)/glucose‐6‐phosphate dehydrogenase (G6PD) axis. Co‐immunoprecipitation, Western blot, and confocal immunofluorescence analyses demonstrate the direct binding interaction between HA‐c‐Raf and Myc‐HSP90, as well as between Flag‐G6PD and Myc‐HSP90. Meanwhile, the overexpression of HSP90 disrupts the benefits of sorafenib on hepatic IRI, and inhibition of G6PD also rescues its protective effect. Notably, in human liver transplant recipients, elevated HSP90 levels correlated with poor graft function and survival, supporting its clinical relevance. Furthermore, a novel oral nanoparticle delivery system, targeting the liver tissue, enhances the therapeutic efficacy of sorafenib, restoring liver enzyme levels by up to 76%. Collectively, these findings identify middle‐dose sorafenib, particularly when delivered via the novel oral nanoplatform, as an effective strategy to mitigate hepatic IRI.

## Graphical abstract

Proposed mechanism of middle‐dose sorafenib modulates redox homeostasis via c‐Raf/HSP90/G6PD axis to attenuate hepatic IRI. This study demonstrates that middle‐dose sorafenib mitigates hepatic IRI through the c‐Raf/HSP90/G6PD axis to enhance pentose phosphate pathway‐mediated antioxidant defense and redox homeostasis, thereby alleviating inflammatory responses and reducing apoptosis.

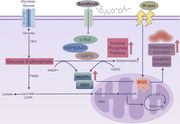

## 1 Introduction

Liver transplantation (LT) is one of the most effective treatments for patients with end‐stage liver disease [1, 2]. Hepatic ischemia reperfusion injury (IRI) is common in LT and a major cause of posttransplant graft injury [3, 4]. IRI has been reported to be associated with biliary complications, vascular complications, and delayed liver function recovery, affecting the prognosis of recipients [5, 6]. Hepatic IRI encompasses an initial ischemic period and the subsequent reperfusion period. The ischemic phase induces cellular hypoxic injury and the accumulation of reactive oxygen species (ROS) [7]. During the subsequent reperfusion phase, activation of oxidative stress, infiltration of inflammatory cells, recruitment of inflammatory factors, and accumulation of metabolites collectively exacerbate hepatocyte apoptosis and necrosis [8, 9]. Despite extensive efforts to attenuate IRI, including ischemic preconditioning and diverse pharmacologic interventions [10, 11, 12, 13], the key mechanisms of hepatic IRI remain incompletely defined. To date, no clinically effective strategy is currently available to prevent or treat hepatic IRI.

IRI is increasingly recognized as a metabolic disease process in which rapid metabolic reprogramming critically shapes redox homeostasis and inflammatory responses, ultimately determining the extent of tissue injury and patient survival [14, 15]. A prior analysis indicated that IRI progression exhibits stage‐dependent performance, shifting from early glucose metabolic changes to intermediate inflammatory activation and late lipid metabolic remodeling [16]. Redox homeostasis during metabolic reprogramming plays a pivotal role in the pathogenesis of IRI. The pentose phosphate pathway (PPP) branches from glycolysis to generate ribose‐5‐phosphate and nicotinamide adenine dinucleotide phosphate (NADPH), which maintain cellular redox balance and fuel anabolic reactions [17]. A previous study reported that the diversion of glycolytic flux toward the PPP served as a critical adaptive mechanism to maintain redox homeostasis and support cellular antioxidant defenses in response to renal IRI [18]. However, the mechanisms regulating redox homeostasis in hepatic IRI remain incompletely understood, and effective pharmacologic interventions have yet to be identified.

Sorafenib is an important therapeutic agent for the treatment of unresectable hepatocellular carcinoma (HCC). As an effective inhibitor of multiple kinases, it can inhibit tumor cell proliferation by blocking the Rat sarcoma virus oncogene (Ras)/rapidly accelerated fibrosarcoma (Raf)/mitogen‐activated protein kinase (MEK)/extracellular signal‐regulated kinase (ERK) signaling pathway [19, 20, 21]. In clinical practice, the recommended oral schedule (400 mg, twice per day) of sorafenib is commonly associated with marked adverse effects, including fatigue, loss of appetite, diarrhea, rash, and peeling of the skin [22, 23]. These adverse reactions limit the clinical tolerance of patients and impair beneficial outcomes. However, sorafenib at specific drug concentrations has been reported to preserve cellular physiological functions and even reverse certain pathological changes. Studies have found that middle‐dose sorafenib can inhibit cell necrosis and promote cell proliferation. In vivo experiments have also shown that middle‐dose sorafenib protects against tumor necrosis factor‐induced systemic inflammatory response syndrome and renal IRI [24, 25]. In addition, another study reported that low‐dose sorafenib can reverse hepatic steatosis by promoting mitochondrial uncoupling [26]. Sorafenib was also reported to reverse liver fibrosis by inducing ferroptosis of hepatic stellate cells through the hypoxia‐inducible factor 1‐alpha (HIF‐1α)/solute carrier family 7 member 11 (SLC7A11) axis [27]. However, no study has reported the potential mechanism underlying the protective effects of specific concentrations of sorafenib on hepatic IRI.

In this study, we report the efficacy of sorafenib as a new therapeutic strategy for hepatic IRI. Remarkably, middle‐dose sorafenib (50 mg/kg) can protect hepatocytes from IRI through the inhibition of inflammation and apoptosis. Through the multi‐omics analyses and rescue experiments, our study demonstrates that middle‐dose sorafenib ameliorates hepatic IRI by remodeling the redox state via the c‐Raf/heat shock protein 90 (HSP90)/glucose‐6‐phosphate dehydrogenase (G6PD) axis. It should also be noted that the therapeutic benefit can be further improved through the novel oral nanoparticle delivery system. These findings provide new insights into therapeutic strategies to alleviate hepatic IRI.

## 2 Results

### 2.1 Suitable Concentration of Sorafenib Improves Hepatic IRI by Inhibiting Apoptosis and Inflammation

**FIGURE 1:**
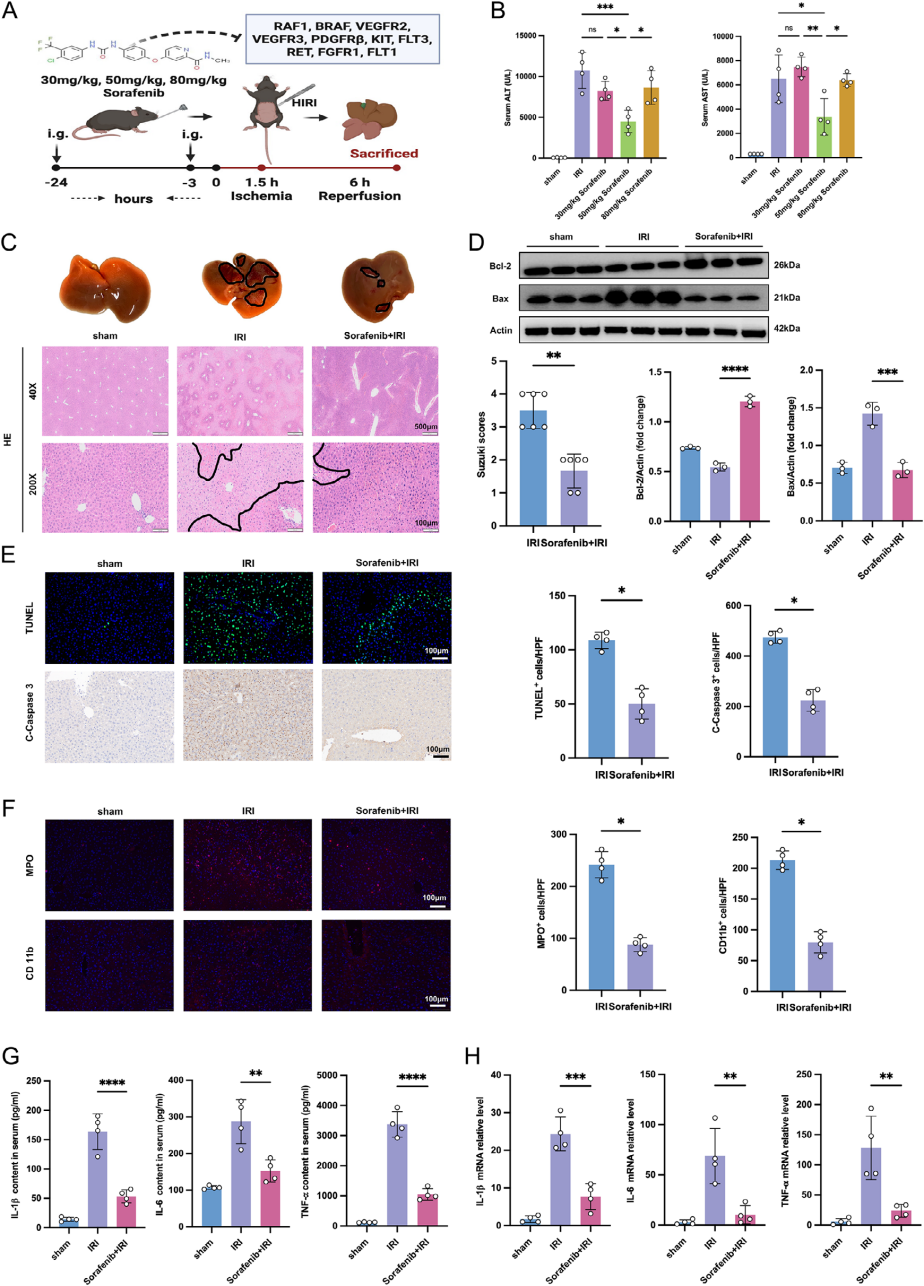
Suitable concentration of sorafenib improves hepatic IRI by inhibiting apoptosis and inflammation. (A) Schematic diagram of hepatic IR operation and sorafenib administration strategy. Mice were subjected to 1.5 h of ischemia followed by 6 h of reperfusion. Mice were treated with different doses of sorafenib 24 and 1.5 h before the ischemia started via oral gavage (i.g.) (*n* = 4/group). (B) Levels of serum ALT and AST in the indicated groups. (C) Representative H&E photographs of sham, IRI, and Sorafenib+IRI groups and their quantifications. (D) Western blot analysis of Bax and Bcl‐2 expression in the liver from the indicated groups (*n* = 3/groups) and quantification of the western blot analysis. (E) Representative TUNEL staining and IHC staining of cleaved‐caspase 3 expression of liver tissues from the indicated groups and their quantifications. (F) Representative MPO and CD11b IF staining (red) in the liver section of the indicated groups and their quantifications. (G) Serum levels of inflammatory factors (IL‐1β, IL‐6, and TNF‐α) in the indicated groups. (H) RT‐qPCR analysis of the mRNA levels of IL‐1β, IL‐6, and TNF‐α in the indicated groups. All data are shown as means ± SDs. ns indicates no significant difference between the specific groups; *p < 0.05, **p < 0.01, ***p < 0.001, ****p < 0.0001.

To assess the protective effects of sorafenib, the hepatic ischemia‐reperfusion model was established using male C57BL/6 mice (no immune‐compromised procedures). Animals were stratified into five groups, including sham (control group), IRI, and sorafenib intervention (30, 50, and 80 mg/kg sorafenib) groups. The livers of mice were subjected to 1.5 h of ischemia followed by 6 h of reperfusion. According to previous studies, mice in the sorafenib intervention groups were treated with different doses of sorafenib by oral gavage 24 and 3 h before further experimental operations (Figure 1A). Serum alanine aminotransferase (ALT) and aspartate aminotransferase (AST) levels in the IRI group were significantly increased when compared with those in the sham group. Furthermore, our study showed that mice treated with sorafenib at 50 mg/kg (defined as the middle‐dose group) exhibited significantly reduced serum ALT and AST levels compared with the IRI group. In contrast, sorafenib at 30 or 80 mg/kg did not improve the liver function (Figure 1B). Moreover, the middle‐dose sorafenib group showed a marked reduction in hepatic necrotic area compared with the IRI group (Figure 1C). In addition, Western blot (WB) analysis revealed increased expression of the antiapoptotic factor B‐cell leukemia/lymphoma (Bcl‐2) and reduced expression of the proapoptotic factor B‐cell leukemia/lymphoma‐associated X protein (Bax) in the livers of the middle‐dose sorafenib group versus the IRI group (Figure 1D). Then we employed cleaved caspase‐3 immunohistochemistry (IHC) staining and terminal deoxynucleotidyl transferase‐mediated deoxyguanosine triphosphate nick‐end labeling (TUNEL) staining to assess the hepatocyte apoptosis. As expected, IRI markedly increased apoptotic death, whereas a middle dose of sorafenib significantly attenuated this apoptosis (Figure 1E).

During the hepatic IRI, inflammation is initiated during the ischemia period and is further exacerbated during the reperfusion period. Therefore, we further explored whether middle‐dose sorafenib could mitigate the hepatic inflammatory response. Immunofluorescence (IF) staining revealed that middle‐dose sorafenib markedly reduced hepatic infiltration of MPO^+^ and CD11b^+^ inflammatory cells compared with the IRI group (Figure 1F). The nuclear factor kappa‐light‐chain‐enhancer of activated B cells (NF‐κB) pathway is a central component of innate immune signaling and is pivotal in orchestrating inflammatory responses during hepatic IRI. Consistent with this, western blot analysis demonstrated that the protein levels of NF‐κB signaling pathway molecules (P‐P65 and inhibitor of nuclear factor kappa B alpha, IκBα) were decreased in the liver of the Sorafenib+IRI group compared with the IRI group (Figure S1A,B). As shown in Figure 1G, serum levels of interleukin‐1 beta (IL‐1β), interleukin‐6 (IL‐6), and tumor necrosis factor‐alpha (TNF‐α) were significantly reduced in the Sorafenib+IRI group compared with the IRI group. Consistently, hepatic tissues from the Sorafenib+IRI group exhibited markedly lower mRNA expression of these pro‐inflammatory cytokines and chemokines (Figure 1H).

### 2.2 Middle‐Dose Sorafenib Inhibits the Expression of HSP90 to Improve Hepatic IRI

**FIGURE 2:**
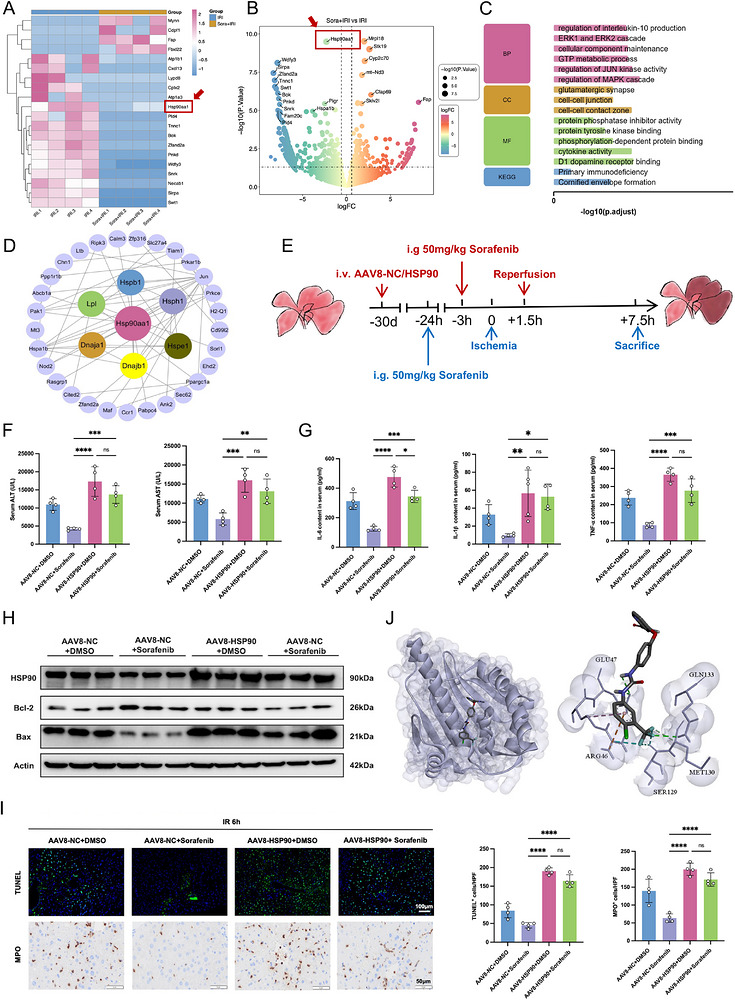
Middle‐dose sorafenib inhibits the expression of HSP90 to improve hepatic IRI. (A–C) Hierarchical clustering heat map, volcano plot, and GO enrichment analysis for the comparison of Sorafenib+IRI and IRI groups. (D) Seven transcriptional factors (Hsp90aa1, Hspe1, Dnajb1, Dnaja1, Lpl, Hspb1, and Hsph1) show the most differentially expressed in the RNA‐seq analysis. (E, F) Levels of serum ALT and AST in the AAV8‐NC+DMSO, AAV8‐NC+sorafenib, AAV8‐HSP90+DMSO, and AAV8‐HSP90+Sorafenib (*n* = 4/group) after hepatic IRI. (G) Serum levels of inflammatory factors (IL‐1β, IL‐6, and TNF‐α) in the indicated groups. (H) Western blot analysis of HSP90, Bax, and Bcl‐2 expression in liver from the indicated groups (*n* = 3/groups) and quantification of the western blot analysis. (I) Representative TUNEL staining and IHC staining of MPO expression of liver tissues from the indicated groups and their quantification. (J) Molecular docking simulation result of sorafenib with the HSP90 protein. All data are shown as means ± SDs. ns indicates no significant difference between the specific groups; *p < 0.05, **p < 0.01, ***p < 0.001, ****p < 0.0001.

To decipher the underlying mechanism of middle‐dose sorafenib on the protection of hepatic IRI, we performed transcriptomic analysis of liver tissues from these three groups of mice. The results of RNA sequencing (RNA‐Seq) showed significant differences in genes between Sorafenib+IRI and the IRI groups (Figure 2A). Volcano plot indicated that there were 427 significantly downregulated differentially expressed genes (DEGs) and 137 upregulated DEGs (*p* < 0.05) (Figure 2B). The Gene Ontology (GO) enrichment analysis showed that IRI significantly upregulated pathways related to inflammation and cytokine activity (Figure 2C). Similarly, transcriptomic profiling identified significant gene‐expression differences between the sham and IRI groups (Figure S2A–C). According to the correlation analysis of transcriptomics, it was found that Hsp90aa1 (HSP90), Hspe1, Dnajb1, etc., were the most differentially expressed transcription factors (Figure 2D).

HSP90 has been implicated in immune regulation and maintenance of tissue metabolism and redox balance. In addition, it also mediates cellular defenses against oxidative stress and plays a regulatory role in cell‐cycle dynamics [28]. In our study, HSP90 was significantly upregulated following IRI and was significantly downregulated by middle‐dose sorafenib. Taken together, these findings suggest that inhibition of HSP90 may contribute to the protective effects. To test our hypothesis, we delivered adeno‐associated virus 8 (AAV8)‐HSP90 via tail‐vein injection to achieve hepatic HSP90 overexpression in mice. Mice receiving the AAV8‐NC served as the controls (Figure 2E). In this part of the experiment, our study reported that serum ALT and AST levels in AAV8‐HSP90 mice were significantly higher than those in AAV8‐NC and AAV8‐NC+Sorafenib mice after IRI. However, no significant differences in ALT or AST levels were observed between the AAV8‐HSP90 and AAV8‐HSP90+Sorafenib groups (Figure 2F). In addition, the Enzyme‐Linked Immunosorbent Assay (ELISA) and Real‐time Quantitative Polymerase Chain Reaction (RT‐qPCR) results also showed a similar tendency (Figure 2G, S2D). Furthermore, both the outcomes of WB analysis and hematoxylin and eosin (HE), myeloperoxidase (MPO), and TUNEL staining of inflammatory and apoptotic proteins showed that the overexpression of HSP90 reversed the protective effects of middle‐dose sorafenib (Figure 2H–I; Figure S2E,F). These results indicate that HSP90 overexpression abolishes the protective effects of middle‐dose sorafenib, confirming that the protective effects of middle‐dose sorafenib are mediated through HSP90.

We further performed molecular docking and dynamics simulations to investigate the interactions between sorafenib and HSP90 proteins. The top 10 docking conformations were scored, and the highest‐ranked binding mode with a favorable interaction energy was selected for detailed analysis. The results showed that sorafenib forms hydrogen bonds and Pi–cation interactions with HSP90, specifically forming hydrogen bonds with the amino acid residues Gln133 and Glu47, and a Pi–cation interaction with Arg46 (Figure 2J). Subsequent studies could investigate the precise amino acid residues involved in sorafenib–protein interactions to more comprehensively elucidate the molecular mechanisms through which sorafenib exerts its protective effects on hepatic IRI.

### 2.3 Middle‐dose Sorafenib Promotes the Activation of the Pentose Phosphate Pathway via Inhibition of HSP90 to Alleviate Oxidative Stress During Hepatic IRI

**FIGURE 3:**
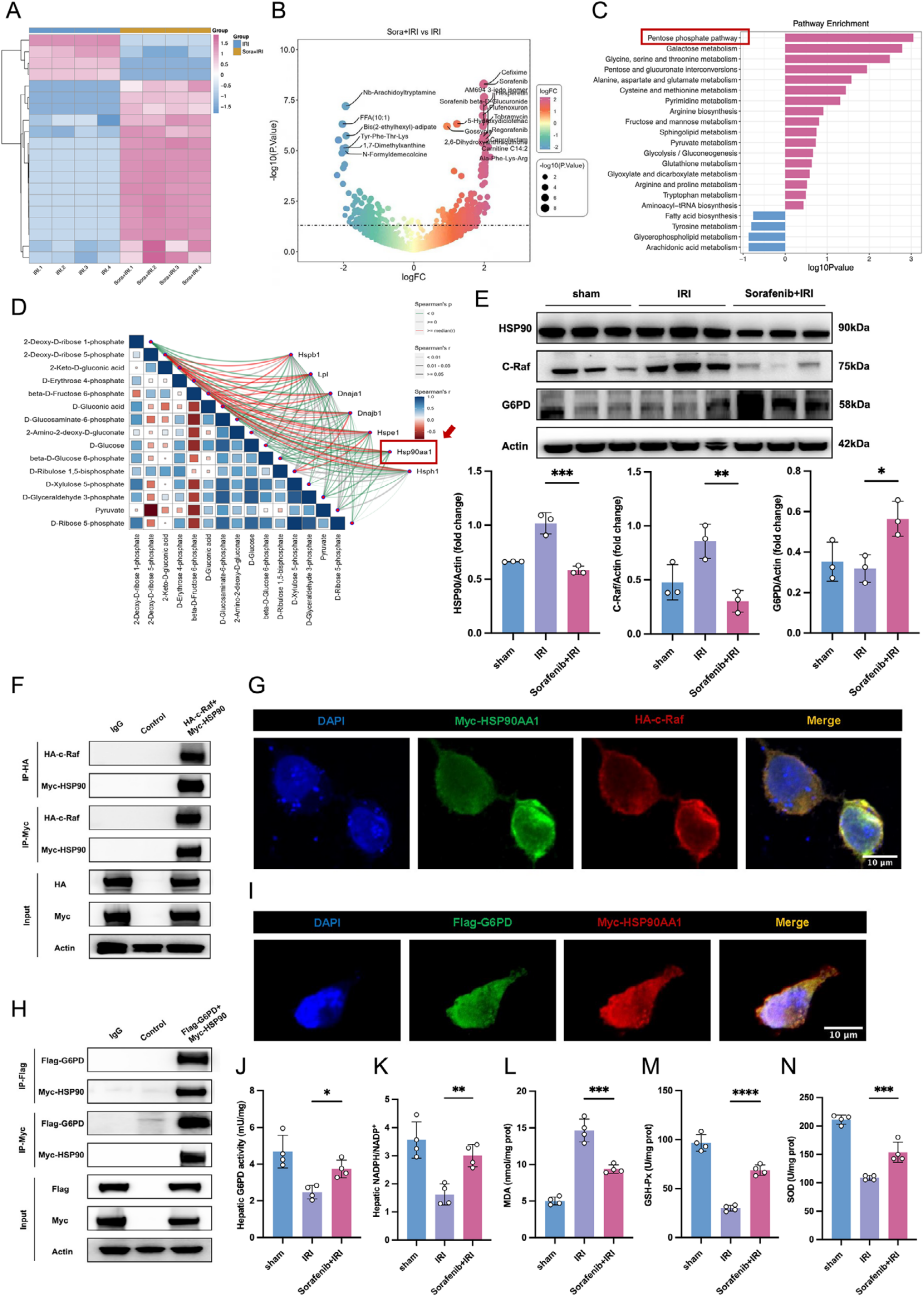
Middle‐dose sorafenib promotes the activation of the pentose phosphate pathway via c‐Raf/HSP90/G6PD axis to alleviate oxidative stress during hepatic IRI. (A) Hierarchical clustering heat maps show the distribution profiles of detected DEMs when compared with IRI and Sorafenib+IRI groups. (B) Volcano plots indicate the DEMs (red, upregulated metabolites; blue, downregulated metabolites) when compared with the IRI and Sorafenib+IRI groups. (C) Pathway‐based analysis of metabolic changes. (D) Using the Mantel test for correlated analysis to jointly examine the seven most significantly different transcription factors and the metabolites of the pentose phosphate pathway. (E) Western blot analysis of HSP90, c‐Raf, and G6PD expression in liver from the sham, IRI, and Sorafenib+IRI groups (*n* = 3/groups) and quantification of the western blot analysis. (F, G) Co‐immunoprecipitation, western blot, and confocal immunofluorescence analyses used to demonstrate the direct interactions between HA–c‐Raf and Myc–HSP90. (H, I) Co‐immunoprecipitation, western blot, and confocal immunofluorescence analyses used to demonstrate the direct interactions between Myc–HSP90 and Flag–G6PD. (J, K) Hepatic G6PD activity and NADPH/NADP^+^ ratio in liver tissues were detected in the indicated groups. (L–N) The MDA level, GSH‐Px level, and SOD level were detected in the indicated groups. All data are shown as means ± SDs. ns indicates no significant difference between the specific groups; *p < 0.05, **p < 0.01, ***p < 0.001, ****p < 0.0001.

Given the central role of rapid metabolic remodeling in hepatic IRI, we performed the metabolomic analysis of liver tissues from these three groups of mice. The principal component analysis (PCA) model was established for clustering metabonomic characteristics, which clearly separated the three groups of samples (Figure S3A). The heatmap analysis indicated substantial metabolomic divergence among sham, IRI, and Sorafenib+IRI groups (Figure 3A; Figure S3B). Volcanic mapping showed that there were 502 differentially expressed metabolites (DEMs) (312 low expression and 190 high expression, *p* < 0.05) among the annotated metabolites when comparing the sham group with the IRI group, and 192 DEMs (147 low expression and 45 high expression, *p* < 0.05) when comparing the Sorafenib+IRI group with the IRI group (Figure 3B; Figure S3C). The GO enrichment analysis revealed that PPP‐associated pathways exhibited the most significant differences across three experimental groups (Figure 3C). Combined with the transcriptomic analysis, the Mantel test was used to analyze the correlation between the most differentially expressed transcription factors and PPP metabolites. It was also worth noting that the highest correlation with PPP metabolites was HSP90 (Figure 3D). This suggests that HSP90 may play an important role in the process of IRI through the PPP. HSP90 is a central regulator of proteostasis, and its ATPase cycle drives conformational transitions of the HSP90 dimer. Formation of the RAF1 (c‐Raf)—HSP90—CDC37 (RHC) complex is essential for the activity of both RAF1 and HSP90 [29]. Therefore, we hypothesized that middle‐dose sorafenib exerts its protective role through c‐Raf/HSP90 to mediate the activation of the PPP. Our WB results showed higher HSP90 and c‐Raf protein levels in the IRI group and lower levels in the Sorafenib+IRI group, while G6PD protein levels were significantly higher in the Sorafenib+IRI group when compared with the IRI group (Figure 3E). To gain deeper insight into the direct protein–protein interactions among c‐Raf, HSP90, and G6PD, we subsequently performed the following experiments. Co‐immunoprecipitation (Co‐IP) and WB analyses were then performed to demonstrate the direct interactions between HA‐c‐Raf and Myc‐HSP90, as well as between Myc‐HSP90 and Flag‐G6PD, confirming pairwise protein–protein associations. Furthermore, confocal immunofluorescence analysis validated the direct interactions among these proteins (Figure 3F–I). We further examined the expression levels of c‐Raf, HSP90, and G6PD in mice treated with 30 and 80 mg/kg sorafenib before IRI. The results showed that none of these proteins exhibited significant changes in either treatment group (Figure S3D). In vitro experiments further demonstrated that HSP90 protein levels were significantly suppressed following sorafenib treatment at 2 and 5 µM, whereas G6PD protein levels remained unchanged (Figure S3E). These findings suggest that sorafenib may primarily affect the PPP through modulation of G6PD enzymatic activity rather than its protein abundance.

As we all know, G6P can be diverted into the oxidative branch of the PPP to generate NADPH, a key component that sustains cellular antioxidant defenses. In our study, we observed a significant decline in G6PD activity in the IRI group. However, the activity of G6PD was significantly restored in the Sorafenib+IRI group (Figure 3J). The altered redox state correlated with changes in the NADPH/NADP^+^ ratio (Figure 3K). Malondialdehyde (MDA), a lipid peroxidation product, together with antioxidant indices such as superoxide dismutase (SOD) and glutathione peroxidase (GSH‐Px), are commonly used to evaluate ROS‐related oxidative stress. Consistent with this, IRI increased hepatic MDA levels and reduced SOD and GSH‐Px activities compared with the sham group, whereas middle‐dose sorafenib could reverse these effects (Figure 3L–N). Further molecular docking and molecular dynamics simulation analyses revealed that sorafenib interacted with G6PD through hydrogen bonding, Pi–Pi, Pi–anion, and Pi–cation interactions, including hydrogen bonds with Tyr401, Arg393, and Gln372, Pi–Pi interactions with Trp509 and Tyr507, as well as Pi–anion and Pi–cation interactions with Glu417 and Lys403, respectively (Figure S3F).

### 2.4 6‐Aminonicotinamide (6‐AN), a G6PD Suppressor, Reversed the Protective Effects of Middle‐Dose Sorafenib in Hepatic IRI

**FIGURE 4:**
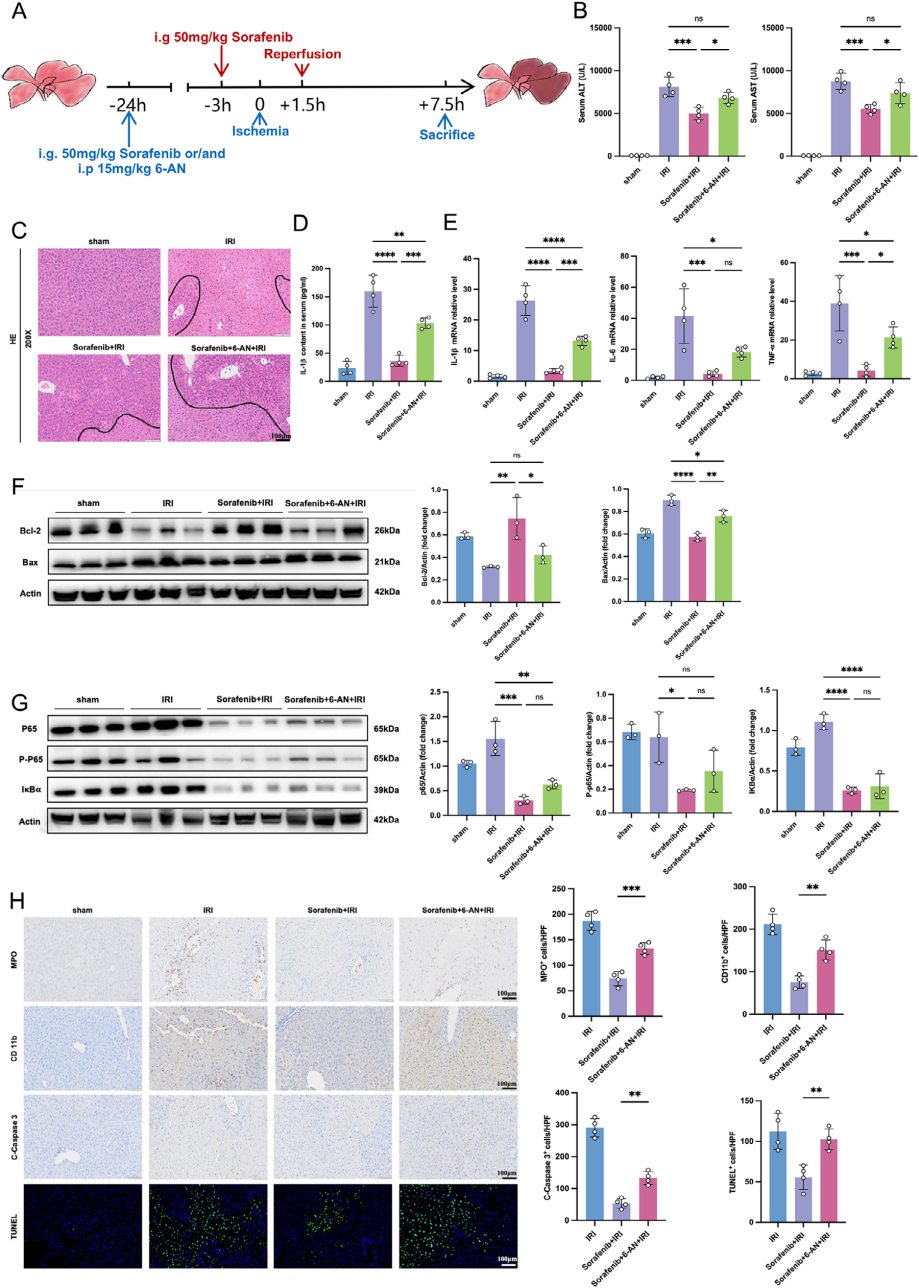
The G6PD suppressor 6‐Aminonicotinamide (6‐AN) reverses the protective effects of middle‐dose sorafenib on hepatic IRI. (A, B) Levels of serum ALT, AST in the sham, IRI, sorafenib+IRI, and sorafenib+6‐AN+IRI groups (*n* = 4/group). (C) Representative HE staining of the indicated groups. (D) Serum levels of inflammatory factors (IL‐1β) in the indicated groups. (E) RT‐qPCR analysis of the mRNA levels of IL‐1β, IL‐6, and TNF‐α in the indicated groups. (F) Western blot analysis of Bax and Bcl‐2 expression in the liver from the indicated groups (*n* = 3/groups) and quantification of the western blot analysis. (G) Protein levels of P‐P65 and IκBα in the indicated groups and quantification of the western blot analysis. (H) Representative IHC staining of MPO, CD11b, cleaved‐caspase 3 expression, and TUNEL staining of liver tissues from the indicated groups and their quantification. All data are shown as means ± SDs. ns indicates no significant difference between the specific groups; *p < 0.05, **p < 0.01, ***p < 0.001, ****p < 0.0001.

To further evaluate whether the protective impact of middle‐dose sorafenib on hepatic IRI was PPP‐dependent, we pharmacologically inhibited PPP activity in vivo using 6‐AN, a selective G6PD inhibitor (Selleck; CAS 329‐89‐5), as previously described [9]. Accordingly, mice were administered 6‐AN or vehicle (dissolved in dimethyl sulfoxide, DMSO) before IRI and were assigned to four groups, including sham, IRI, Sorafenib+IRI, and Sorafenib+6‐AN+IRI (Figure 4A). Importantly, 6‐AN largely abolished middle‐dose sorafenib‐induced alleviation of hepatic damage after 6 h postreperfusion, showing that the Sorafenib+6‐AN+IRI group had significantly higher ALT and AST levels when compared with the Sorafenib+IRI group (Figure 4B). Consistently, HE staining showed that the Sorafenib+6‐AN+IRI group had a significantly larger hepatic necrotic area than the Sorafenib+IRI group (Figure 4C). Moreover, pharmacological inhibition of G6PD blunted the middle‐dose sorafenib‐induced reduction in circulating proinflammatory cytokines and their associated protein levels in serum (Figure 4D,E). Moreover, our WB results showed that cellular apoptosis and proinflammatory responses were promoted by 6‐AN in the Sorafenib+6‐AN+IRI group (Figure 4F,G). It was also worth noting that the inhibition of G6PD led to a significant increase in proinflammatory response as well as cellular death and apoptosis, as evidenced by the outcomes of MPO, CD11b, cleaved caspase‐3, and TUNEL staining (Figure 4H).

### 2.5 Pharmacological Administration of Sorafenib via the Oral Nanoparticle Delivery System Further Attenuated Hepatic IRI

**FIGURE 5:**
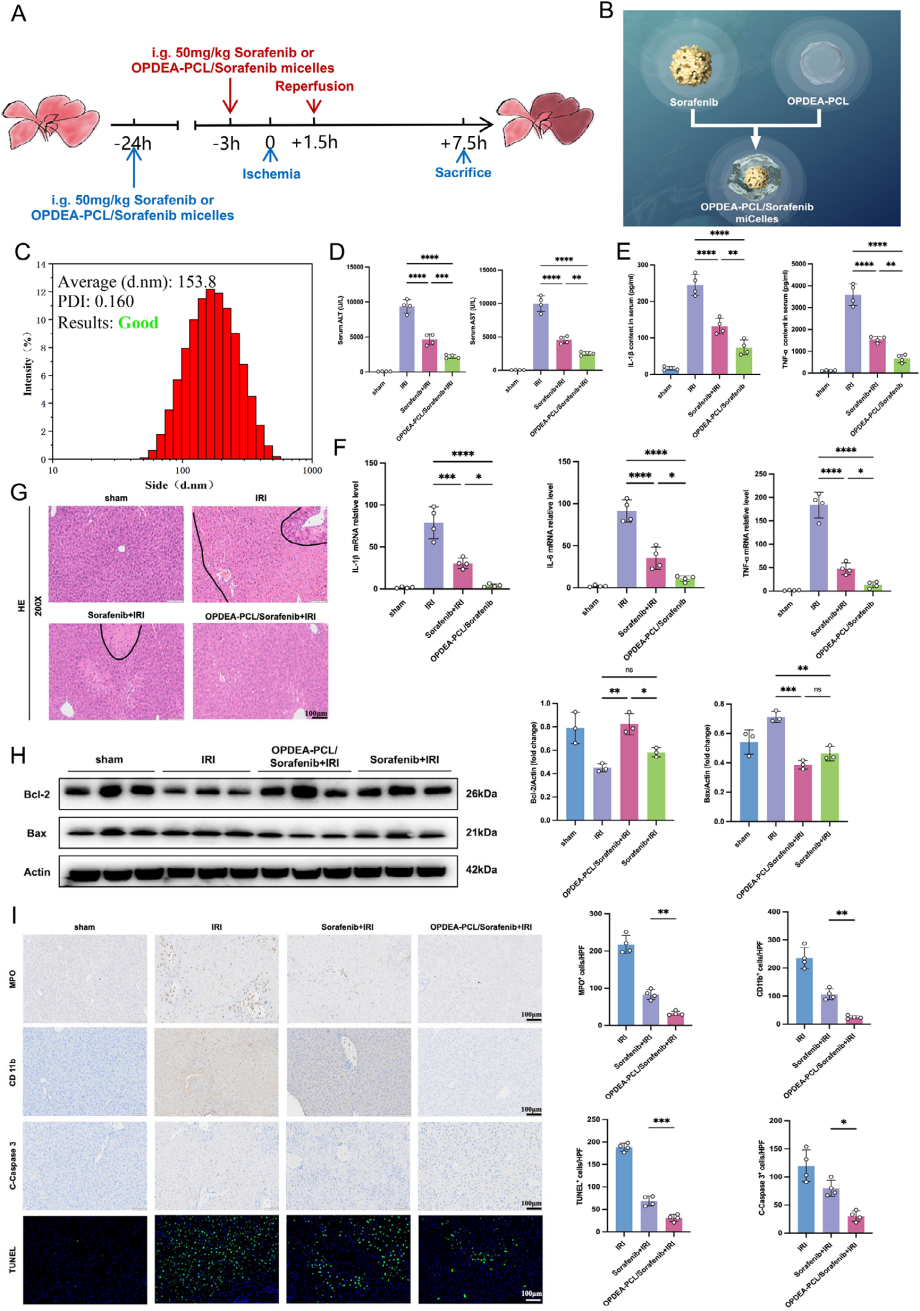
Attenuation of hepatic IRI by the pharmacological supply of middle‐dose sorafenib based on the novel nanoparticle delivery system. (A, B) The chemical structure and synthesis process of sorafenib nanoparticle. (C) The particle size and zeta potential of the nanomedicine. (D) Levels of serum ALT, AST in the sham, IRI, Sorafenib+IRI, and OPDEA‐PCL/Sorafenib+IRI (*n* = 4/group). (E) Serum levels of inflammatory factors (IL‐1β and TNF‐α) in the indicated groups. (F) RT‐qPCR analysis of the mRNA levels of IL‐1β, IL‐6, and TNF‐α in the indicated groups. (G) Representative HE staining of the indicated groups. (H) Western blot analysis of Bax and Bcl‐2 expression in the liver from the indicated groups (*n* = 3/groups) and quantification of the western blot analysis. (I) Representative IHC staining of MPO, CD11b, cleaved‐caspase 3 expression, and TUNEL staining of liver tissues from the indicated groups and their quantification. All data are shown as means ± SDs. ns indicates no significant difference between the specific groups; *p < 0.05, **p < 0.01, ***p < 0.001, ****p < 0.0001.

To improve liver‐targeted delivery and therapeutic protection, we developed a straightforward and safe platform that increases hepatic sorafenib accumulation using a novel oral nanoparticle delivery. OPDEA‐PCL/Sorafenib micelles were synthesized and used as shown in Figure 5A,B. The OPDEA‐PCL/Sorafenib micelles exhibited an average particle size of 153.8 nm and a polydispersity index (PDI) of 0.160, indicating favorable uniformity (Figure 5C). To assess the in vivo accumulation pattern of the novel oral nanoparticle‐based delivery system, we conducted live imaging studies. Consistent with our design, the imaging results demonstrated that the formulation preferentially accumulated in the liver (Figure S4A,B).

The livers of mice in the OPDEA‐PCL/Sorafenib group exhibited markedly attenuated hepatic injury compared with those treated with sorafenib alone. This improvement was evidenced by significant reductions in serum biochemical markers of liver injury and pro‐inflammatory cytokine production following IRI (Figure 5D–F). In addition, WB, IHC staining, and TUNEL staining results showed that OPDEA‐PCL/Sorafenib preconditioning resulted in markedly less hepatocellular necrosis and apoptosis, as well as reduced inflammatory cell infiltration, compared with Sorafenib alone after IRI (Figure 5G–I; Figure S4C).

### 2.6 HSP90 Expression Was Closely Correlated With Postoperative Liver Function and Survival Outcomes of LT Recipients

**FIGURE 6:**
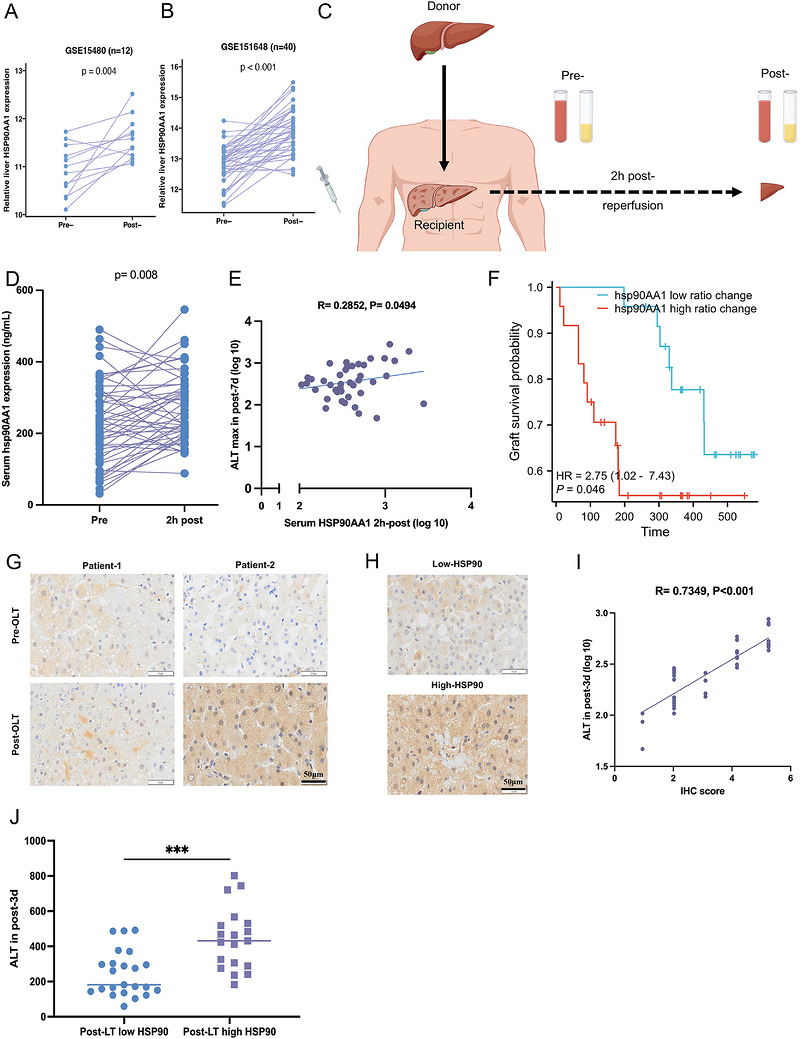
HSP90 expression was correlated with postoperative liver function and survival outcomes of liver transplant recipients. (A, B) HSP90 mRNA levels in 12 and 40 donor livers of persons subjected to OLT from the GSE15480 and GSE151648 databases, respectively. (C) Diagram of obtaining blood and liver tissue samples from liver transplant recipients. (D) Comparison of pre‐ and post‐LT HSP90 levels in the serum (*n* = 48). (E) The correlation of the serum HSP90 level 2 h postreperfusion and the maximal ALT level within 7 days after transplantation (*p* = 0.0494). (F) According to the change ratio of serum HSP90 (postreperfusion/pretransplant), the 48 recipients were divided into the HSP90 low ratio change and HSP90 high ratio change groups. The HSP90 low ratio change group has improved graft survival (*p* = 0.046). (G) Representative IHC staining of HSP90 in the liver tissue of patients undergone LT. (H) 48 recipients who underwent LT were divided into low (*n* = 24) and high (*n* = 24) HSP90 expression groups of post‐LT livers using IHC scores (IHC score < 3 as the threshold). (I) Pearson's correlation coefficient between the postreperfusion livers’ IHC scores and serum ALT at post‐3d (*R* ^2^ = 0.7349, *p* < 0.001). (J) The correlation of the post‐LT livers’ IHC scores (post‐LT low HSP90 group and post‐LT high HSP90 group) and the ALT level in the post‐3d (*p* < 0.001). All data are shown as means ± SDs. ns indicates no significant difference between the specific groups; *p < 0.05, **p < 0.01, ***p < 0.001, ****p < 0.0001.

To validate our findings under the LT condition, we analyzed HSP90 expression in liver samples from patients undergoing orthotopic liver transplantation (OLT) using two public datasets: GSE15480 (*n* = 12) and GSE151648 (*n* = 40). The mRNA levels of HSP90 were significantly elevated in reperfusion samples compared with baseline specimens (Figure 6A,B). Furthermore, we analyzed the correlation between the expression of HSP90 in pre‐ and postreperfusion serum and the prognosis of LT in 48 recipients (Figure 6C). Accordingly, we found that the protein level of HSP90 was significantly increased after reperfusion (Figure 6D). We observed that higher postreperfusion serum HSP90 levels were associated with a higher peak ALT within the first 7 days after LT (Figure 6E). According to the change ratio of serum HSP90 (postreperfusion/prereperfusion), these recipients were divided into the HSP90 low ratio change group (*n* = 24) and the HSP90 high ratio change group (*n* = 24). Survival analysis also indicated that the HSP90 low ratio change improved graft survival (*p* = 0.046, Figure 6F). IHC staining confirmed HSP90 expression in liver tissues from patients undergoing LT (Figure 6G). Based on IHC scores (with a threshold of <3), post‐LT liver specimens from 48 recipients were stratified into low (*n* = 24) and high (*n* = 24) HSP90 expression groups (Figure 6H). We found that IHC scores in reperfusion liver were strongly correlated with serum ALT on postoperative day 3 after LT (Figure 6I). Similarly, higher IHC scores of HSP90 protein levels in the postreperfusion liver samples were correlated with higher serum ALT on postoperative day 3 after LT (Figure 6J).

## 3 Discussion

**FIGURE 7:**
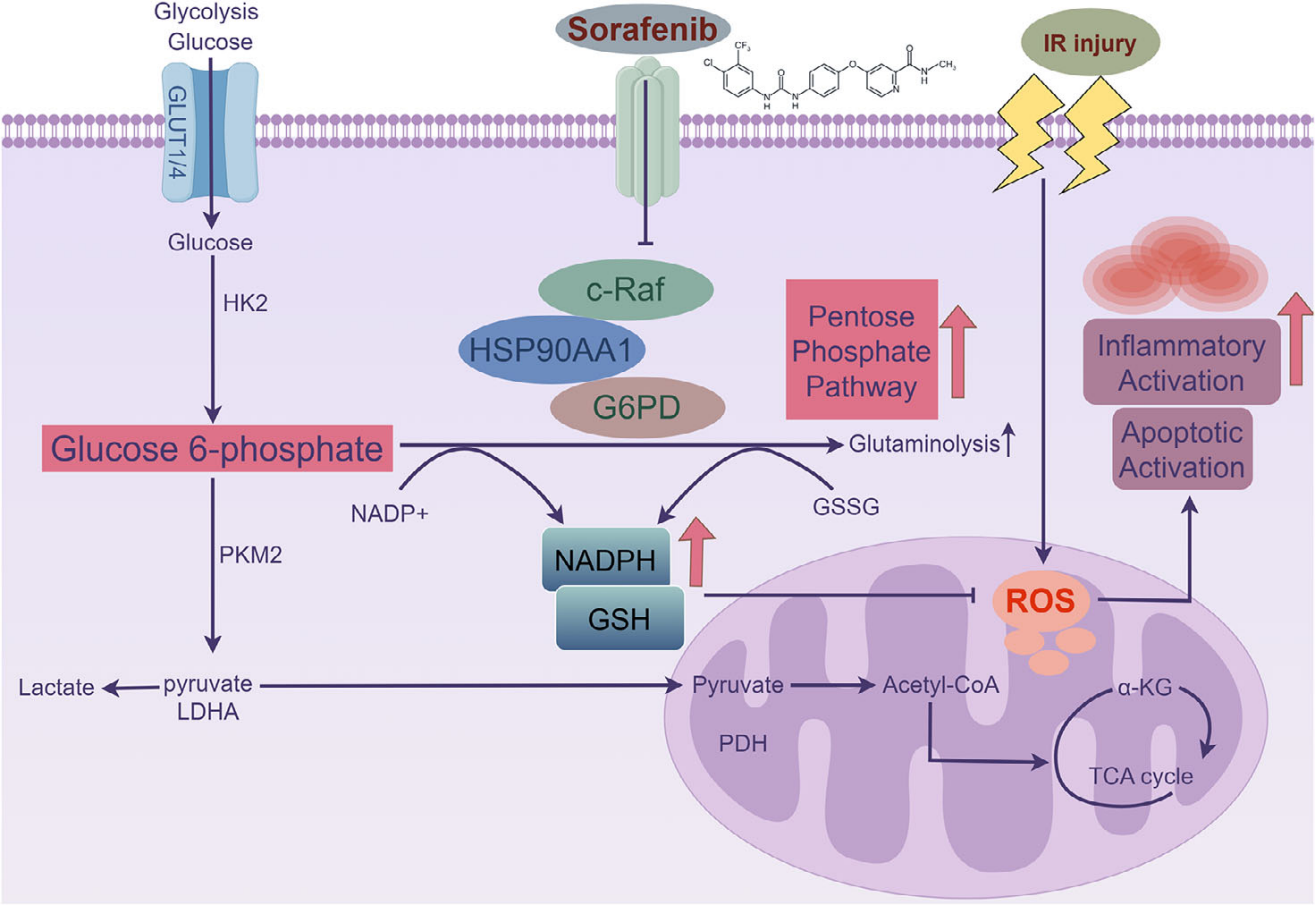
Proposed mechanism of middle‐dose sorafenib modulates redox homeostasis via c‐Raf/HSP90/G6PD axis to attenuate hepatic IRI. This study demonstrates that middle‐dose sorafenib mitigates hepatic IRI by activating the c‐Raf/HSP90/G6PD axis to enhance pentose phosphate pathway‐mediated antioxidant defense and redox homeostasis.

Hepatic IRI is a major contributor to liver failure after LT, and mitigating this injury at the molecular and cellular levels is critical for improving posttransplant outcomes. In this study, we demonstrated that middle‐dose sorafenib confers protection against hepatic IRI by attenuating apoptosis and inflammation in male C57BL/6 mice (no immune‐compromised procedures). Through transcriptomics and metabolomics, we identified the key protective mechanism involving the target gene HSP90 and the PPP. Our experiments demonstrated the important role of the c‐Raf/HSP90/G6PD axis in hepatic IRI. Moreover, we applied a novel oral nanoparticle delivery system to improve the efficiency of middle‐dose sorafenib in hepatic IRI. Finally, we found that HSP90 expression was closely correlated with postoperative liver function in LT, as validated by clinical liver specimens (Figure 7).

As a multitarget tyrosine kinase inhibitor, sorafenib inhibits signal pathways of tumor growth and angiogenesis. Commonly, sorafenib is used as a single‐agent therapy to target HCC, but it is often resistant and toxic [20]. However, a specific dose of sorafenib was reported to play an important role in various areas. Sorafenib displays a dose‐dependent, biphasic activity profile. In both murine and human cell systems, higher concentrations are cytotoxic and promote cell death, whereas lower doses suppress necroptosis. Moreover, sorafenib has been reported to mitigate TNF‐driven systemic inflammatory response syndrome (SIRS) and protect against TNF‐associated renal IRI [24]. Low‐dose sorafenib, a tenth of a percent, has been reported to improve nonalcoholic steatohepatitis (NASH). Mechanistically, independent of its canonical kinase targets in HCC, it exerts its beneficial effects by causing a slight uncoupling of the mitochondria, which in turn triggers the activation of AMP‐activated protein kinase (AMPK) [30]. Sorafenib also exerted antifibrotic effects. Mejias et al. demonstrated that sorafenib attenuated intrahepatic fibrosis and inflammation, suppressed angiogenesis, and improved portal hypertension in a rat model of cirrhosis. Subsequent work further indicated that sorafenib restrained hepatic stellate cell (HSC) proliferation and reduced the production of fibrosis‐associated proteins [31]. Additionally, a specific dose of sorafenib helps to attenuate liver angiogenesis and inflammation [32]. In this study, we investigated the function of middle‐dose sorafenib in a new model of hepatic IRI, revealed its protective and restorative effect on liver function, and identified the optimal dose of 50 mg/kg sorafenib.

Disruption of redox and metabolic homeostasis is a major driver of hepatic IRI. During the ischemia period, the liver experiences metabolic disturbances, including impaired energy metabolism and accumulation of toxic metabolites, which contribute to hepatocyte injury. During the subsequent reperfusion period, the sudden restoration of blood flow generates large amounts of ROS that damage cellular membranes, leading to oxidative stress. In addition to causing direct cellular injury, oxidative stress initiates an inflammatory cascade by activating Kupffer cells and recruiting neutrophils, which further amplify ROS production and release pro‐inflammatory mediators, thereby exacerbating liver damage [33]. Disruption of metabolic homeostasis is a hallmark of hepatic ischemia, exemplified by lipoxygenase overexpression, which drives pathological lipid peroxidation and impairs hepatic fatty acid oxidation [34]. Also, Zhi et al. showed that activation of AMPK alleviated hepatic IRI by modulating energy metabolism, oxidative stress, mitochondrial function, autophagy, inflammatory signaling, and endoplasmic reticulum stress [35]. An imbalance in redox homeostasis, characterized by the disruption of the antioxidant system, was critically governed by the nuclear factor erythroid 2‐related factor 2 (Nrf2) signaling pathway, which coordinately activated hepatic antioxidant defenses and detoxification genes. Moreover, fibroblast growth factor 18 (FGF18) has been shown to alleviate hepatic IRI through the ubiquitin‐specific peptidase 16 (USP16)/Kelch‐like ECH‐associated protein 1 (KEAP1)/Nrf2 signaling axis [36, 37]. Our study aimed to protect against hepatic IRI by precisely targeting the preservation of metabolic and redox homeostasis.

HSP90, a highly conserved molecular chaperone protein, has the function of assisting protein folding, stabilizing protein conformation, participating in protein degradation, and regulating protein activity. HSP90 interacts with multiple regulators in signal transduction, such as protein kinases, transcription factors, and so on [38]. Zhao et al. reported that HSP90 could promote ferroptosis via the glutathione (GSH)/ glutathione peroxidase 4 (GPX4) pathway. Given that ferroptosis contributed to renal injury, they further proposed that HSP90 plays a regulatory role in renal IRI [39]. It has also been reported that the function of HSP90 is involved in IRI in the heart, brain, and kidney. Among these studies, Harrison et al. showed that inhibition of HSP90 was conducive to renal IRI during kidney transplantation in mice [40]. HSP90 plays an important role in maintaining tissue metabolism, immune response, and redox homeostasis. The conformational cycle of HSP90 is tuned through its interactions with a range of co‐chaperones, including Hop (Hsp70‐Hsp90 organizing protein), CDC37, p23, AHA1 (activator of Hsp90 ATPase homolog 1), and protein phosphatase 5 (PP5), thereby affecting malignant tumor progression [41, 42]. In addition, HSP90 is involved in the cellular response to oxidative stress and affects cell cycle regulation, particularly through the modulation of the cell cycle regulatory protein cyclin B1, a key substrate of the ubiquitin–proteasome system [28]. Our study showed that there was a relationship between HSP90 and PPP metabolites. Mechanically, based on the pathway correlation between sorafenib and HSP90, our study demonstrated that middle‐dose sorafenib played a protective role through c‐Raf/HSP90 to mediate the activation of the PPP.

The pentose phosphate pathway plays a wide role in IRI across multiple organs. Xia et al. found that PPP attenuated redox stress and alleviated myocardium IRI [43]. In addition, a study found that mitochondrial ferritin protects against IR‐induced apoptosis by regulating glucose metabolism via the PPP in cerebral IRI [44]. Roelofs et al. reported that the PPP was the only metabolic pathway upregulated at 24 h after renal IRI, potentially conferring oxidative protection and supporting cellular regeneration [18]. In an ischemia‐free liver transplantation procedure, He et al. found that the antioxidant PPP remained active. In contrast, this pathway was significantly suppressed during cold storage but underwent rapid and sustained reactivation following reperfusion [45]. In this study, we found that the PPP was the most significantly altered pathway through the analysis of metabolomics, indicating that middle‐dose sorafenib played a key role in PPP during hepatic IRI. As we all know, G6PD is the only rate‐limiting enzyme of the PPP [46]. G6PD deficiency is closely related to acute hemolytic disease. In this study, we used 6‐aminonicotinamide as a G6PD inhibitor to suppress PPP activity. This intervention reversed the protective effects of middle‐dose sorafenib and resulted in more severe hepatic IRI.

Hepatic IRI is a multifactorial process characterized by microvascular dysfunction, oxidative stress, activation of innate and adaptive immunity, and diverse forms of programmed cell death (e.g., apoptosis, pyroptosis, and ferroptosis), which limits the efficacy of single‐target pharmacological interventions [47]. Moreover, many potential therapeutic agents require effective concentrations to be reached either before ischemia or at the beginning period of reperfusion, making clinical translation difficult due to the complexity and variability of donor and recipient conditions [48]. Recent studies indicated that hypothermic oxygenated perfusion (HOPE) and normothermic machine perfusion (NMP), two widely used machine perfusion approaches, substantially reduce the risk of early graft dysfunction and biliary complications after transplantation [49, 50]. These approaches represented effective strategies for mitigating IRI [51]. In the future, the combination of machine perfusion with intelligent material‐based drug delivery systems and the optimization of perfusate composition may offer a promising strategy to further attenuate hepatic IRI and achieve clinical translation.

We acknowledge that the current study has some limitations. It is necessary to validate the direct interaction of middle‐dose sorafenib with HSP90 more specifically. Further experimental validation is required to confirm the computational molecular docking results and to more clearly elucidate the specific mechanisms through which sorafenib exerts its protective effects. Notably, this study applied a nanoparticle delivery system to improve the drug delivery efficiency of middle‐dose sorafenib. However, the safety effects of nanoparticles, such as cytotoxicity and immunotoxicity, still need to be considered and tested in the long term.

## 4 Conclusion

In summary, this study demonstrates that middle‐dose sorafenib mitigates hepatic IRI via the c‐Raf/HSP90/G6PD axis to enhance PPP‐mediated antioxidant defense and redox homeostasis. These findings identify middle‐dose sorafenib as a potential therapeutic agent for hepatic IRI, with liver‐targeted nanoparticle delivery further enhancing its efficacy and translational potential.

## 5 Materials and Methods

### 5.1 Animals

All male mice were housed under specific pathogen‐free conditions at 23 ± 2°C with a 12 h light/dark cycle. Experimental animals were 8–10 weeks of age and weighed 25 ± 2 g on average. Nutritional needs were supplied by both food and water. All mice were purchased from the Zhejiang Center of Laboratory Animals (Zhejiang, China). Animal procedures were conducted in compliance with the ARRIVE 2.0 reporting guidelines to ensure transparent and reproducible research. The study protocol was reviewed and approved by the Institutional Animal Care and Use Committee of the Zhejiang Center of Laboratory Animals (approval no. ZJCLA‐IACUC‐20020140). All procedures were designed in accordance with the 3Rs to minimize suffering, reduce animal use, and refine endpoints when possible.

### 5.2 Mouse Hepatic IRI Model

Mice were subjected to a standard 70% partial hepatic IRI procedure. After induction of anesthesia with pentobarbital sodium (50 mg/kg), the portal triad was transiently occluded using microvascular clips at a site above the bifurcation toward the right lateral lobe, thereby restricting inflow to the left lateral and median lobes. After 90 min of ischemia, the clips were removed to initiate 6 h of reperfusion. Sham animals underwent the same operative steps without portal triad occlusion. Mice were kept on a heating pad set to 37°C until they were fully awake. Subsequently, serum and liver tissue were collected for further experiments.

### 5.3 Liver Biochemical Measurement

Serum ALT and AST levels were determined on a Chemray 800 analyzer (Shenzhen, China). Systemic inflammation was evaluated by measuring serum cytokines with uncoated mouse ELISA kits (Invitrogen): IL‐6 (88‐7064‐88), IL‐1β (88‐7013‐88), and TNF‐α (88‐7324‐22).

### 5.4 Histological and Immunohistochemical Staining

Liver specimens were fixed in 10% formalin, processed through graded dehydration, paraffin‐embedded, and cut into 5 µm sections. Sections were stained with HE to evaluate the degree of hepatic necrosis. IHC was performed to assess Myeloperoxidase (MPO) (ab208670; Abcam), Cluster of Differentiation 11b (CD11b; CY5019; Abways), cleaved caspase3 (#9664S; Cell Signaling Technology), and HSP90 (#4874S; Cell Signaling Technology) expression. IHC staining intensity was independently scored by two experienced pathologists using a semi‐quantitative scale: 0 (negative), 1 (weak), 2 (weak to moderate), 3 (moderate), 4 (moderate to strong), and 5 (strong). Representative images were acquired on an Olympus IX83 microscope.

### 5.5 Immunofluorescence (IF) and TUNEL Staining

IF staining and confocal imaging were performed as follows: 293T cells were seeded onto sterile glass coverslips and transfected with plasmids expressing HA‐tagged c‐Raf, Myc‐tagged HSP90, and Flag‐tagged G6PD for 24 h. Then, the cells were rinsed with phosphate‐buffered saline (PBS), fixed in 4% paraformaldehyde (PFA) prepared in PBS for 15 min at room temperature, and then permeabilized with 0.1% Triton X‐100 in PBS for 10 min. After blocking with 5% BSA in PBS for 1 h to reduce nonspecific binding, samples were incubated overnight at 4°C with primary antibodies diluted in 1% BSA/PBS: anti‐Myc (60003‐2‐Ig, Proteintech), anti‐HA (51064‐2‐AP, Proteintech), and anti‐Flag (66008‐4‐Ig, Proteintech). After extensive washing, cells were then incubated at room temperature for 1 h with species‐matched, fluorophore‐conjugated secondary antibodies corresponding to the green and red fluorescence channels, and nuclei were counterstained with 4',6‐diamidino‐2‐phenylindole (DAPI, ServiceBio, #G1012). Coverslips were mounted with an antifade reagent and imaged on a laser‐scanning confocal microscope using an oil‐immersion objective. Furthermore, to assess cell apoptosis and inflammatory responses in the liver tissue slides, TUNEL staining (ServiceBio, #G1502) was performed, along with the application of primary antibodies targeting mouse MPO (ab208670; Abcam) and CD11b (CY5019; Abways).

### 5.6 Assessment of G6PD Activity and Cellular Redox Status

Commercial kits from Beyotime and Solarbio (China) were used to evaluate various parameters in hepatic tissues. These assessments included measuring G6PD activity, MDA levels, NADPH/NADP^+^ levels, total SOD activity, and GSH‐Px activity.

### 5.7 Real‐Time Quantitative Polymerase Chain Reaction

Liver tissues were processed for total ribonucleic acid (RNA) extraction with the total RNA isolation kit (Yeason, China). RNA concentration was measured on a NanoDrop instrument (Thermo Fisher Scientific) and reverse‐transcribed to complementary deoxyribonucleic acid (cDNA) using SuperMix for qPCR (Yeason). Quantitative PCR was then carried out with SYBR Green chemistry (qPCR SYBR Green Master Mix; Yeason). Relative mRNA levels were calculated after normalization to β‐actin. Primer information (Tsingke, Wuhan, China) is listed in Table S1.

### 5.8 WB Analysis

Total protein was isolated using an immunoprecipitation assay lysis buffer (Fdbio Science) supplemented with a protease inhibitor cocktail, phosphatase inhibitors, and phenylmethylsulfonyl fluoride (PMSF; Fdbio Science). Protein concentrations were quantified using a BCA protein assay kit (Fdbio Science). The protein supernatants were then mixed with 5× sodium dodecyl sulfate (SDS) loading buffer and denatured at 95°C for 10 min. Proteins were resolved on 10% sodium dodecyl sulfate polyacrylamide gel electrophoresis (SDS‐PAGE) gels and transferred onto polyvinylidene fluoride membranes (IPVH00010; Millipore). Membranes were blocked in 5% skim milk for 90 min and then incubated with primary antibodies at 4°C overnight. After primary antibody incubation, membranes were probed with the corresponding secondary antibodies. Chemiluminescent signals were detected using a ChemiDoc MP imaging system, and β‐actin was used as the loading control. Antibody information for Western blotting is summarized in Table S2.

### 5.9 AAV8 Vectors Construction and Intravenous Delivery in Mice

The hepatocyte‐specific AAV8 vector for HSP90 overexpression was generated by Vigene Biosciences Co., Ltd. (Shandong, China). Then mice received a lateral tail‐vein injection of AAV8‐TBG‐HSP90 at 1.5 × 10^11^ genome copies. The AAV8‐GFP vector was administered as the control. Four weeks after viral delivery, mice underwent hepatic IR surgery.

### 5.10 Transcriptomics and Metabolomics Analysis

Liver tissues were obtained from the sham, IRI, and Sorafenib+IRI groups. Sample processing and extraction for both transcriptomic and metabolomic sequencing were performed by Metware Biotechnology Co., Ltd. (Wuhan, China) [33]. For transcriptomic profiling, cDNA libraries were sequenced on an Illumina platform. For the metabolomics study, metabolites were identified using the Metware database. PCA was employed to reveal the intrinsic differences in the data. Genes and metabolites exhibited a fold change greater than 2.0 and a *p*‐value < 0.05 between the two groups, were classified as DEGs and DEMs. The Kyoto Encyclopedia of Genes and Genomes (KEGG) and GO were utilized for pathway enrichment analysis of differential genes.

### 5.11 Nanoparticle Delivery System

To improve treatment efficacy, we formulated sorafenib into an OPDEA‐PCL nanoparticle platform that enables more effective delivery [52]. In summary, sorafenib was dissolved in DMSO to obtain a concentration of 25 mg/mL sorafenib solution. OPDEA‐PCL material was dissolved in acetonitrile (ACN) to obtain a solution of OP at a concentration of 10 mg/mL. The above sorafenib solution and OPDEA‐PCL solution were mixed according to the mass ratio of 1:5. The mixture of material and drug was stirred on a magnetic stirrer at room temperature. At the same time, an equal volume of deionized water was added to the magnetic stirring for half an hour, and then the solution was removed and placed in dialysis bags. Then they were placed in ionic water for dialysis and renewed every 0.5 h. OPDEA‐PCL/Sorafenib nanomedicine was obtained by removing the solution from the dialysis bag after 2 h of dialysis. Particle size distribution and zeta potential were measured in 10 mM HEPES (pH 7.4) with a DLS analyzer (Zetasizer Nano ZS90; Malvern Instruments, Malvern, UK).

### 5.12 Human Liver Tissues

Liver samples were collected from recipients undergoing orthotopic liver transplantation (OLT) at Shulan (Hangzhou) Hospital. Left‐lobe tissue was obtained at two time points, including cold storage and 2 h after portal reperfusion periods. Written informed consent was obtained from all participants. The study was approved by the Ethics Committee of Shulan (Hangzhou) Hospital (No. KY2023029) and was conducted in accordance with the ethical principles of the Declaration of Helsinki.

### 5.13 Statistical Analysis

All continuous data are expressed as mean ± standard deviation (SD), while categorical data are presented as frequencies or counts. Statistical analysis was carried out using GraphPad Prism 9.0 software (GraphPad Software Inc., La Jolla, CA, USA). For data that followed a normal distribution, comparisons between two groups were made using the unpaired two‐tailed Student's *t*‐test, while differences among multiple groups were evaluated through one‐way analysis of variance (ANOVA) followed by relevant post hoc tests. Nonnormally distributed data were analyzed using the Wilcoxon test. Additionally, Pearson's correlation and a general linear regression model were employed to determine the association between serum HSP90 levels and biochemical variables in the human serum and histological datasets. The significance levels were represented as *p < 0.05, **p < 0.01, ***p < 0.001, and ****p < 0.0001.

## Author Contributions

Xiao Xu and Kai Wang designed the whole project. Fengqiang Gao, Libin Dong, and Yawen Tan performed most of the experiments, manuscript writing, and data analysis of the study. Zhen Zhang, Shengjun Xu, and Zhengxing Lian helped with in vivo experiments. Fengqiang Gao, Kai Wang, and Xiao Xu revised the manuscript. Zijian Lou and Yichao Wu helped with liver transplant data acquisition. Shusen Zheng and Li Zhuang helped with the provision of liver tissues for liver transplant recipients. Nasha Qiu and Siyu Chen helped with the synthesis of nanomedicine. Xiao Xu and Kai Wang were responsible for manuscript editing and revision, and provided scientific research funding support. All authors have read and approved the final manuscript.

## Funding

This work was supported by The National Key Research and Development Program of China (No. 2021YFA1100500, 2024YFA1107200); General Program of National Natural Science Foundation of China (No. 82470683); High‐Level Talent Recruitment and Cultivation Program of Jiangsu Province Hospital (The First Affiliated Hospital of Nanjing Medical University) (No. 2025 (EV25)).

## Ethics Statement and Consent to Participate

The experimental protocols were authorized by the Institutional Review Board of the Institutional Animal Care and Use Committee, Zhejiang Center of Laboratory Animals (No. ZJCLA‐IACUC‐20020140). The Ethics Committee of Shulan (Hangzhou) Hospital gave approval for this research, which was conducted in accordance with the ethical principles of the 1975 Declaration of Helsinki (No. KY2023029).

## Conflicts of Interest

The authors declare no conflicts of interest.

## Supporting information




**Supporting Figure 1**: Suitable concentration of sorafenib improves hepatic IRI by inhibiting apoptosis and inflammation. (A) Protein levels of NF‐κB signaling pathway molecules (P‐P65 and IκBα) in the liver of the sham, IRI, and sorafenib+IRI groups. (B) Quantification of the western blot analysis. The data are shown as means ± SDs. ns indicates no significant difference between the specific groups; *p < 0.05, **p < 0.01, ***p < 0.001, ****p < 0.0001.
**Supporting Figure 2**: Low‐dose sorafenib inhibits the expression of HSP90 to improve hepatic IRI. (A–C) Hierarchical clustering heat map, volcano plot, and GO enrichment analysis of RNA‐seq for the comparison of sham and IRI groups. (D) RT‐qPCR analysis of the mRNA levels of IL‐1β, IL‐6, and TNF‐α in the indicated groups in the AAV8‐NC+DMSO, AAV8‐NC+Sorafenib, AAV8‐HSP90+DMSO, and AAV8‐HSP90+Sorafenib (n = 4/group) groups after hepatic IRI. (E) Representative HE staining of the indicated groups. (F) Quantification of the western blot analysis. The data are shown as means ± SDs. ns indicates no significant difference between the specific groups; *p < 0.05, **p < 0.01, ***p < 0.001, ****p < 0.0001.
**Supporting Figure 3**: Low‐dose sorafenib promotes the activation of the pentose phosphate pathway via c‐Raf/HSP90/G6PD axis to alleviate oxidative stress during hepatic IRI. (A) Individual PCA image shows the global sample distribution profiles (*n* = 4/group). (B) Hierarchical clustering heat maps show the distribution profiles of detected DEMs compared with sham and IRI groups. (C) Volcano plots indicate the DEMs (red, upregulated metabolites; blue, downregulated metabolites) compared with the sham and IRI groups. (D) Western blot analysis of HSP90, c‐Raf, and G6PD expression in the liver from the IRI, 30mg/kg Sorafenib, and 80mg/kg Sorafenib groups (*n* = 4/groups) and quantification of the western blot analysis. (E) Western blot analysis of HSP90 and G6PD expression in the AML12 cell lines after treatment with different concentrations of sorafenib. (F) Molecular docking simulation result of sorafenib with the G6PD protein. The data are shown as means ± SDs. ns indicates no significant difference between the specific groups; *p < 0.05, **p < 0.01, ***p < 0.001, ****p < 0.0001.
**Supporting Figure 4**: Attenuation of hepatic IRI by the pharmacological supply of low‐dose sorafenib based on the novel nanoparticle delivery system. (A, B) In vivo fluorescence imaging of mice at 12 and 24 h following oral administration of OPDEA‐PCL/Sorafenib/DiR (*n* = 3/groups). (C) Western blot analysis of P‐P65 and IκBα expression in liver from the indicated groups (*n* = 3/groups) and quantification of the western blot analysis. The data are shown as means ± SDs. ns indicates no significant difference between the specific groups; *p < 0.05, **p < 0.01, ***p < 0.001, ****p < 0.0001.
**Supporting Table 1**: Primers for real‐time qPCR detection.
**Supporting Table 2**: Antibodies for immunoblot analyses.

## Data Availability

All data generated or analyzed during this study are included in this published article.
