# Supplementary material for: Sorafenib Restores Pentose Phosphate Pathway‐Related Redox Homeostasis via the c‐Raf/HSP90/G6PD Axis in Hepatic Ischemia‐Reperfusion Injury
Source: MedComm (2020). 2026 Jun 21;7(7):e70819. doi: 10.1002/mco2.70819 (PMC13284494; doi:10.1002/mco2.70819)
Supplement: Supplementary file 1 — Supporting Figure 1: Suitable concentration of sorafenib improves hepatic IRI by inhibiting apoptosis and inflammation. (A) Protein levels of NF‐κB signaling pathway molecules (P‐P65 and IκBα) in the liver of the sham, IRI, and sorafenib+IRI groups. (B) Quantification of the western blot analysis. The data are shown as means ± SDs. ns indicates no significant difference between the specific groups; *p < 0.05, **p < 0.01, ***p < 0.001, ****p < 0.0001. Supporting Figure 2: Low‐dose sorafenib inhibits the expression of HSP90 to improve hepatic IRI. (A–C) Hierarchical clustering heat map, volcano plot, and GO enrichment analysis of RNA‐seq for the comparison of sham and IRI groups. (D) RT‐qPCR analysis of the mRNA levels of IL‐1β, IL‐6, and TNF‐α in the indicated groups in the AAV8‐NC+DMSO, AAV8‐NC+Sorafenib, AAV8‐HSP90+DMSO, and AAV8‐HSP90+Sorafenib (n = 4/group) groups after hepatic IRI. (E) Representative HE staining of the indicated groups. (F) Quantification of the western blot analysis. The data are shown as means ± SDs. ns indicates no significant difference between the specific groups; *p < 0.05, **p < 0.01, ***p < 0.001, ****p < 0.0001. Supporting Figure 3: Low‐dose sorafenib promotes the activation of the pentose phosphate pathway via c‐Raf/HSP90/G6PD axis to alleviate oxidative stress during hepatic IRI. (A) Individual PCA image shows the global sample distribution profiles (n = 4/group). (B) Hierarchical clustering heat maps show the distribution profiles of detected DEMs compared with sham and IRI groups. (C) Volcano plots indicate the DEMs (red, upregulated metabolites; blue, downregulated metabolites) compared with the sham and IRI groups. (D) Western blot analysis of HSP90, c‐Raf, and G6PD expression in the liver from the IRI, 30mg/kg Sorafenib, and 80mg/kg Sorafenib groups (n = 4/groups) and quantification of the western blot analysis. (E) Western blot analysis of HSP90 and G6PD expression in the AML12 cell lines after treatment with different concentra [file MCO2-7-e70819-s001.docx]

**Supplementary data**

**Title: Sorafenib restores pentose phosphate pathway-related redox homeostasis via the c-Raf/HSP90/G6PD axis in hepatic ischemia-reperfusion injury**

**Running title: Sorafenib protects against hepatic injury**

Fengqiang Gao ^1,†^, Libin Dong ^2,†^, Yawen Tan ^1,†^, Zhen Zhang ^3,†^, Shengjun Xu ^4^, Zijian Lou ^2^, Yichao Wu ^5^, Siyu Chen ^4^, Li Zhuang ^6^, Zhengxing Lian ^4^, Shusen Zheng ^6^, Nasha Qiu ^4,*^, Kai Wang ^5,*^, Xiao Xu ^7,2,*^

**Author affiliations**

**1** Zhejiang University School of Medicine, Hangzhou 310058, China

**2** Institute of Translational Medicine, Zhejiang University School of Medicine, Hangzhou 310000, China

**3** Department of Orthopedics, The First Affiliated Hospital of Zhejiang University School of Medicine, Hangzhou 310003, China

**4** Department of Hepatobiliary & Pancreatic Surgery, Xihu University School of Medicine, Affiliated Hangzhou First People’s Hospital, Hangzhou 310014, China

**5** Department of Hepatobiliary & Pancreatic Surgery and Minimally Invasive Surgery, Zhejiang Provincial People's Hospital (Affiliated People's Hospital), Hangzhou Medical College, Hangzhou 314408, China

**6** Department of Hepatobiliary and Pancreatic Surgery, Shulan (Hangzhou) Hospital, Hangzhou 310000, China

**7** Hepatobiliary Center, the First Aﬃliated Hospital of Nanjing Medical University, Nanjing 210000, China

^†^These authors have contributed equally to this work and share first authorship.

***Correspondence to:** Prof. Xiao Xu, Hepatobiliary Center, the First Aﬃliated Hospital of Nanjing Medical University, Nanjing 210000, China. *E-mail address*: [zjxu@zju.edu.cn](mailto:zjxu@zju.edu.cn)

Dr. Kai Wang, Department of Hepatobiliary & Pancreatic Surgery and Minimally Invasive Surgery, Zhejiang Provincial People's Hospital (Affiliated People's Hospital), Hangzhou Medical College, Hangzhou 314408, China. *E-mail address:* [kaiw3@zju.edu.cn](mailto:kaiw3@zju.edu.cn)

Dr. Nasha Qiu, Department of Hepatobiliary & Pancreatic Surgery, Xihu University School of Medicine, Affiliated Hangzhou First People’s Hospital, Hangzhou 310014, China. *E-mail address:* [qiunasha@163.com](mailto:qiunasha@163.com)

**Supplementary Figures**

**
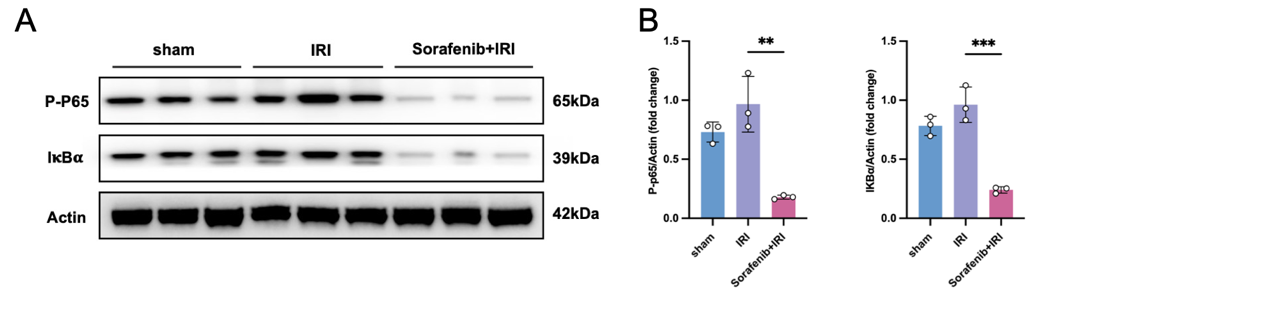
**

**Figure S1.** Suitable concentration of sorafenib improves hepatic IRI by inhibiting apoptosis and inflammation. (A) Protein levels of NF-κB signaling pathway molecules (P-P65 and IκBα) in the liver of the sham, IRI, and sorafenib+IRI groups. (B) Quantification of the western blot analysis. The data are shown as means ± SDs. ns indicates no significant difference between the specific groups; *P< 0.05, **P< 0.01, ***P< 0.001, ****P< 0.0001

**
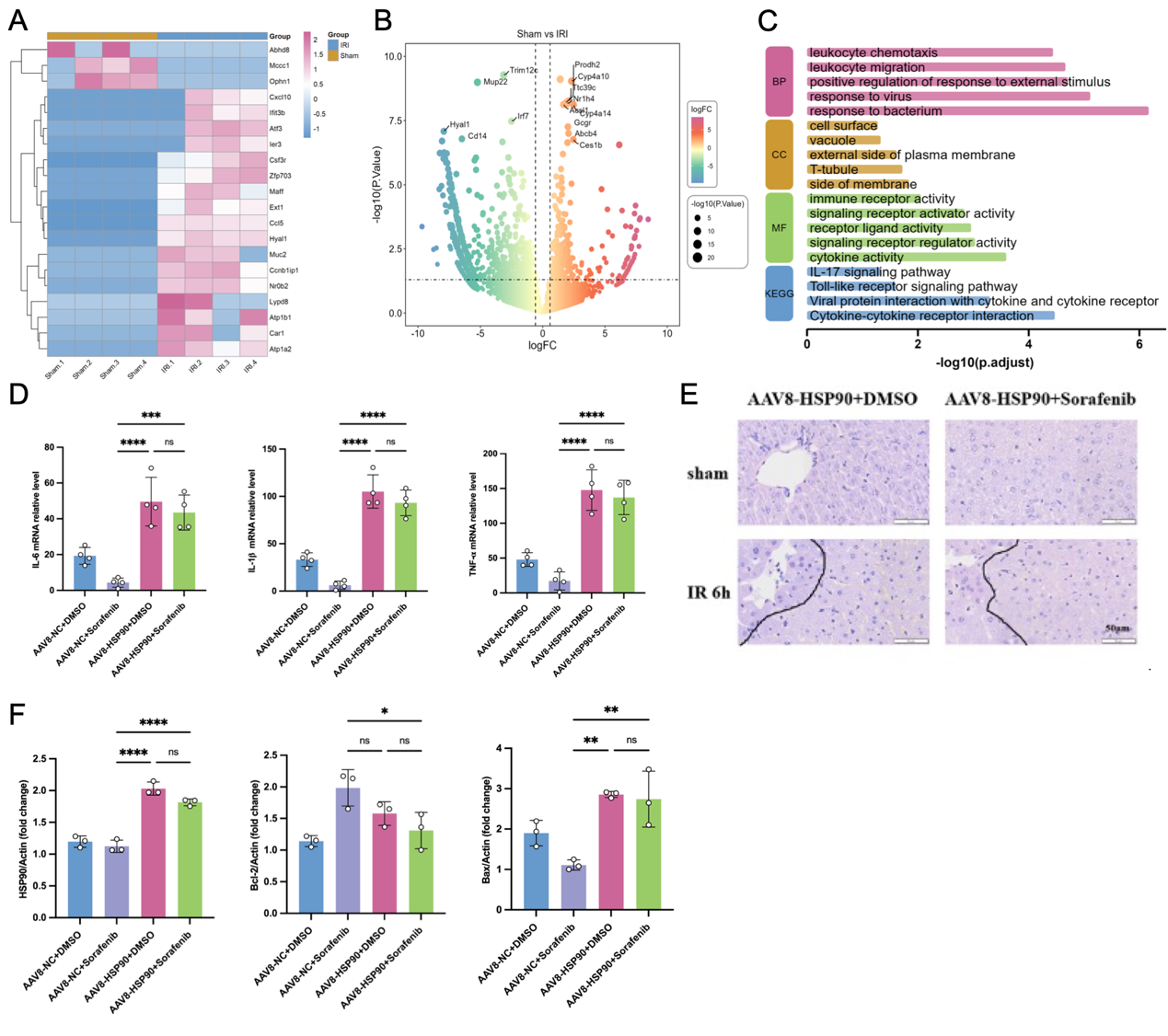
**

**Figure S2.** Low-dose sorafenib inhibits the expression of HSP90 to improve hepatic IRI. (A-C) Hierarchical clustering heat map, volcano plot, and GO enrichment analysis of RNA-seq for the comparison of sham and IRI groups. (D) RT-qPCR analysis of the mRNA levels of IL-1β, IL-6, and TNF-α in the indicated groups in the AAV8-NC+DMSO, AAV8-NC+Sorafenib, AAV8-HSP90+DMSO, and AAV8-HSP90+Sorafenib (n=4/group) groups after hepatic IRI. (E) Representative HE staining of the indicated groups. (F) Quantification of the western blot analysis. The data are shown as means ± SDs. ns indicates no significant difference between the specific groups; *P< 0.05, **P< 0.01, ***P< 0.001, ****P< 0.0001

**
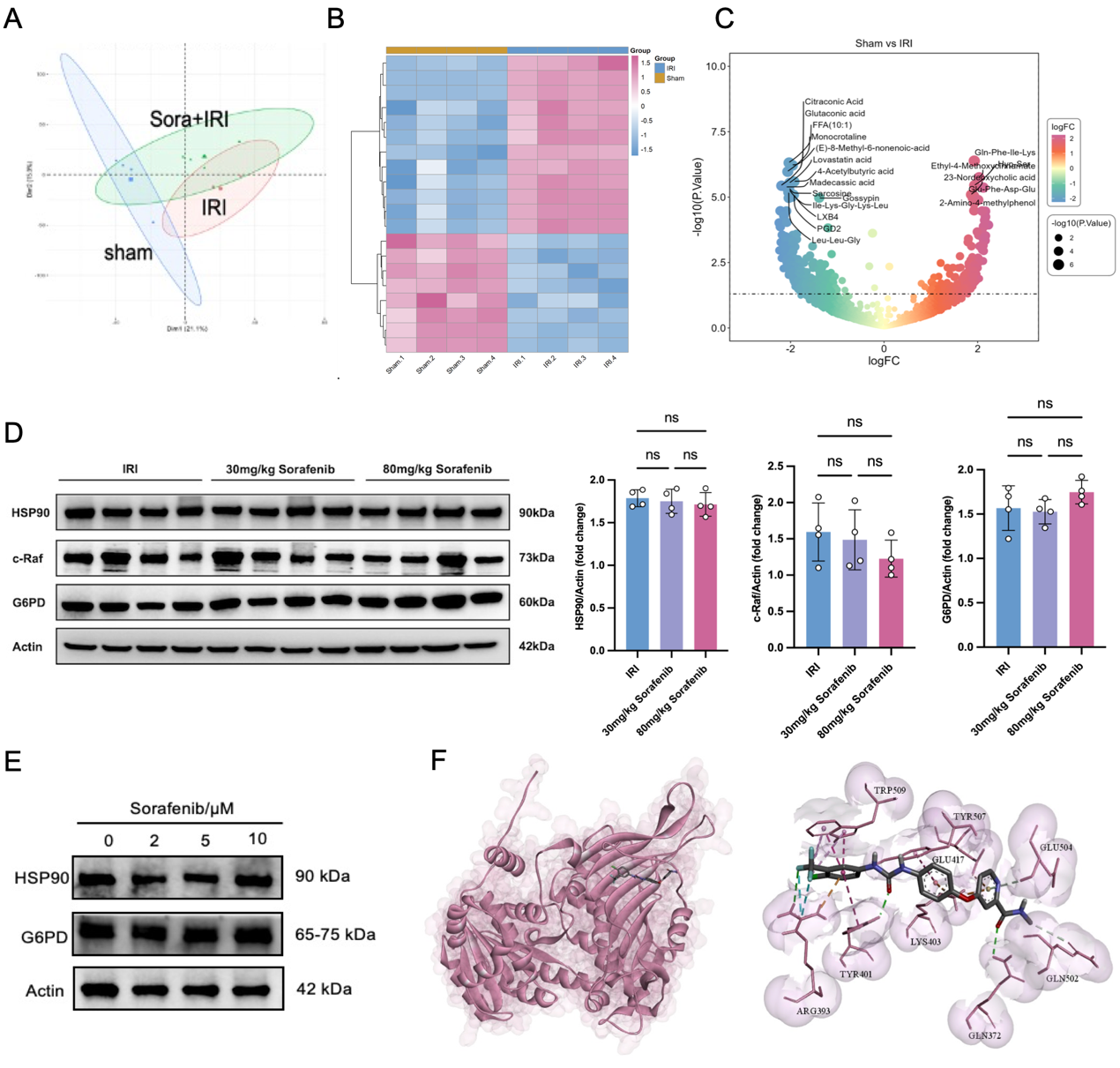
**

**Figure S3.** Low-dose sorafenib promotes the activation of the pentose phosphate pathway via c-Raf/HSP90/G6PD axis to alleviate oxidative stress during hepatic IRI. (A) Individuals PCA image shows the global sample distribution profiles (n=4/group). (B) Hierarchical clustering heat maps show the distribution profiles of detected DEMs compared to sham and IRI groups. (C) Volcano plots indicate the DEMs (red, up-regulated metabolites; blue, down-regulated metabolite) compared to sham and IRI groups. (D) Western blot analysis of HSP90, c-Raf and G6PD expression in the liver from the IRI, 30mg/kg Sorafenib and 80mg/kg Sorafenib groups (n=4/groups) and quantification of the western blot analysis. (E) Western blot analysis of HSP90 and G6PD expression in the AML12 cell lines after treatment with different concentrations of sorafenib. (F) Molecular docking simulation result of sorafenib with the G6PD protein. The data are shown as means ± SDs. ns indicates no significant difference between the specific groups; *P< 0.05, **P< 0.01, ***P< 0.001, ****P< 0.0001

**
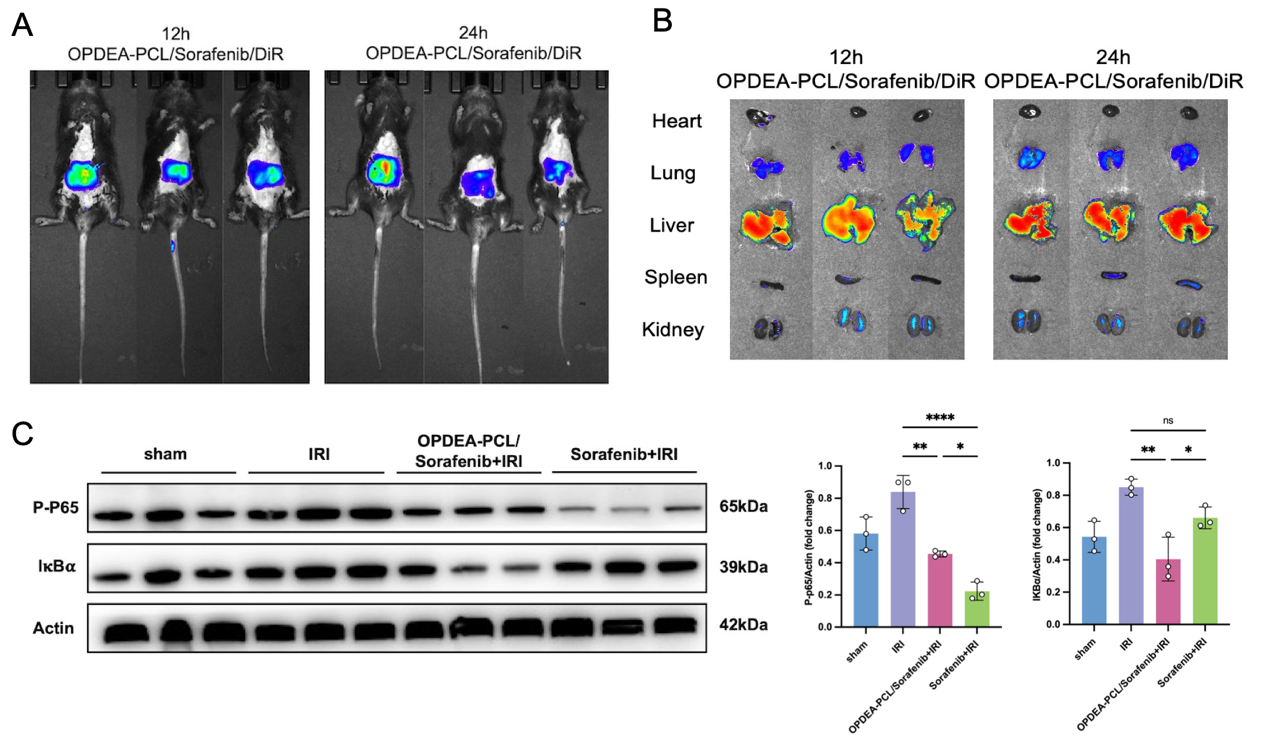
**

**Figure S4.** Attenuation of hepatic IRI by the pharmacological supply of low-dose sorafenib based on the novel nanoparticle delivery system. (A-B) In vivo fluorescence imaging of mice at 12 h and 24 h following oral administration of OPDEA-PCL/Sorafenib/DiR (n=3/groups). (C) Western blot analysis of P-P65 and IκBα expression in liver from the indicated groups (n=3/groups) and quantification of the western blot analysis. The data are shown as means ± SDs. ns indicates no significant difference between the specific groups; *P< 0.05, **P< 0.01, ***P< 0.001, ****P< 0.0001

**Supplementary Tables**

**Table S1. Primers for real-time qPCR detection**

| **Gene** |  | **Sequence5'---3'** |
| --- | --- | --- |
| **Mouse IL-6** | **F** | CTGCAAGAGACTTCCATCCAG |
|  | **R** | AGTGGTATAGACAGGTCTGTTGG |
| **Mouse IL-1β** | **F** | TTCAGGCAGGCAGTATCACTC |
|  | **R** | GAAGGTCCACGGGAAAGACAC |
| **Mouse TNF-α** | **F** | CAGGCGGTGCCTATGTCTC |
|  | **R** | CGATCACCCCGAAGTTCAGTAG |
|  | **R** | CCCCACCGAACTCAAAGAAGG |
| **Mouse** **β-actin** | **F** | GTGACGTTGACATCCGTAAAGA |
|  | **R** | GCCGGACTCATCGTACTCC |

**Table S2. Antibodies for immunoblot analyses**

| **Antibody** | **Cat No.** | **Manufacture** |
| --- | --- | --- |
| **HSP90** | 4874S | CST |
| **Bax** | 2772S | CST |
| **Bcl2** | ab182858 | Abcam |
| **C-Raf** | 9422S | CST |
| **G6PD** | 8866S | CST |
| **β-actin** | 66009-1-Ig | Proteintech |
| **Flag** | 66008-4-Ig | Proteintech |
| **Myc** | 60003-2-Ig | Proteintech |
| **HA** | 51064-2-AP | Proteintech |
